# Iatrogenic aortocoronary arteriovenous fistula

**DOI:** 10.1007/s12471-018-1223-0

**Published:** 2019-01-08

**Authors:** Y. Saleh, A. Almaghraby, B. Hammad, O. Abdelkarim, M. Abdelnaby, K. Herzallah, T. Elzawawy

**Affiliations:** 1 Michigan State University, East Lansing, MI USA; 2 Faculty of Medicine, Alexandria University, Alexandria, Egypt

A 35-year-old female patient with a history of dyslipidaemia and diabetes mellitus presented with exertional angina experienced in the past 2 years. Coronary angiography revealed a proximal left anterior descending (LAD) tight lesion and proximal right coronary artery (RCA) total occlusion. Coronary artery bypass grafting (CABG) was performed, grafting the left internal mammary artery (LIMA) to the LAD and a saphenous venous graft to the RCA. Unfortunately, her symptoms remained unchanged postoperatively. A repeat coronary angiogram 6 months after the CABG revealed a patent LIMA graft to the great cardiac vein draining into the coronary sinus (Fig. 1a and b, Videos 1, 2) and a proximal LAD tight lesion (Fig. 1c, Video 3). A drug-eluting stent was successfully deployed in the LAD (Fig. 1d, Video 4). In our opinion, the LIMA grafted to the great cardiac vein was not haemodynamically significant; hence no intervention was attempted. Subsequently the patient has become asymptomatic.

**Fig. 1:**
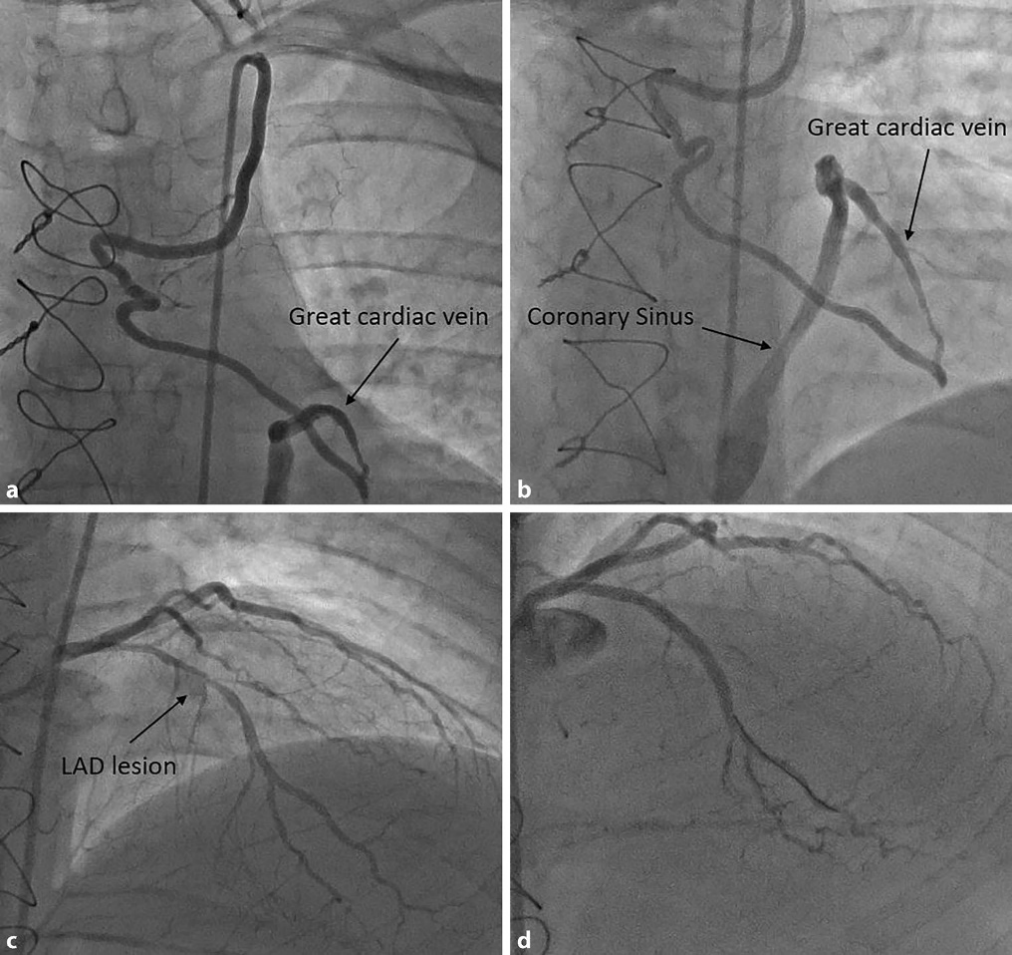
**a** Coronary angiogram showing a left internal mammary artery grafted into the great cardiac vein which drains into the coronary sinus. **b** Coronary angiogram showing a left internal mammary artery grafted into the great cardiac vein which drains into the coronary sinus. **c** Coronary angiogram showing a tight lesion in the left anterior descending artery. **d** Coronary angiogram showing a widely patent stent in the left anterior descending artery

Iatrogenic aortocoronary arteriovenous fistula (ACAVF) is an unusual complication of CABG that results from grafting an artery to a cardiac vein. Coiling or balloon embolisation to occlude the fistula, combined with stenting of the ungrafted artery, is the standard approach in symptomatic patients [1]. In our case, stenting of the ungrafted vessel without occlusion of the ACAVF yielded good results.

## Caption Electronic Supplementary Material

Video 1 Coronary angiogram showing a left internal mammary artery grafted into the great cardiac vein which drains into the coronary sinus

Video 2 Coronary angiogram showing a left internal mammary artery grafted into the great cardiac vein which drains into the coronary sinus

Video 3 Coronary angiogram showing a tight lesion in the left anterior descending artery

Video 4 Coronary angiogram showing a widely patent stent in the left anterior descending artery
